# High *Leptospira* Diversity in Animals and Humans Complicates the Search for Common Reservoirs of Human Disease in Rural Ecuador

**DOI:** 10.1371/journal.pntd.0004990

**Published:** 2016-09-13

**Authors:** Veronica Barragan, Jorge Chiriboga, Erin Miller, Sonora Olivas, Dawn Birdsell, Crystal Hepp, Heidie Hornstra, James M. Schupp, Melba Morales, Manuel Gonzalez, Soraya Reyes, Carmen de la Cruz, Paul Keim, Rudy Hartskeerl, Gabriel Trueba, Talima Pearson

**Affiliations:** 1 The Center for Microbial Genetics and Genomics, Northern Arizona University, Flagstaff, Arizona, United States of America; 2 Instituto de Microbiologia, Colegio de Ciencias Biologicas y Ambientales, Universidad San Francisco de Quito, Quito, Ecuador; 3 Informatics and Computing Program, Northern Arizona University, Flagstaff, Arizona, United States of America; 4 Ministerio de Salud Pública, Quito, Ecuador; 5 Instituto Nacional de Salud Pública e Investigación, Guayaquil, Ecuador; 6 Translational Genomics Research Institute, Flagstaff, Arizona, United States of America; 7 Biomedical Research, Royal Tropical Institute (KIT), Amsterdam, The Netherlands; Universidad Peruana Cayetano Heredia, PERU

## Abstract

**Background:**

Leptospirosis is a zoonotic disease responsible for high morbidity around the world, especially in tropical and low income countries. Rats are thought to be the main vector of human leptospirosis in urban settings. However, differences between urban and low-income rural communities provide additional insights into the epidemiology of the disease.

**Methodology/Principal findings:**

Our study was conducted in two low-income rural communities near the coast of Ecuador. We detected and characterized infectious leptospira DNA in a wide variety of samples using new real time quantitative PCR assays and amplicon sequencing. We detected infectious leptospira in a high percentage of febrile patients (14.7%). In contrast to previous studies on leptospirosis risk factors, higher positivity was not found in rats (3.0%) but rather in cows (35.8%) and pigs (21.1%). Six leptospira species were identified (*L*. *borgpetersenii*, *L kirschnerii*, *L santarosai*, *L*. *interrogans*, *L noguchii*, and an intermediate species within the *L*. *licerasiae* and *L*. *wolffii* clade) and no significant differences in the species of leptospira present in each animal species was detected (χ^2^ = 9.89, adj.p-value = 0.27).

**Conclusions/Significance:**

A large portion of the world’s human population lives in low-income, rural communities, however, there is limited information about leptospirosis transmission dynamics in these settings. In these areas, exposure to peridomestic livestock is particularly common and high prevalence of infectious leptospira in cows and pigs suggest that they may be the most important reservoir for human transmission. Genotyping clinical samples show that multiple species of leptospira are involved in human disease. As these genotypes were also detected in samples from a variety of animals, genotype data must be used in conjunction with epidemiological data to provide evidence of transmission and the importance of different potential leptospirosis reservoirs.

## Author Summary

Leptospirosis is a febrile disease responsible for high morbidity rates all around the world. It is caused by spirochete bacteria in the genus *Leptospira* and transmitted to humans through animal urine or contaminated soil or water. The epidemiology of leptospirosis has been extensively studied in urban communities, where rats are thought to be the main animal reservoir of the disease. However, leptospirosis has been under-reported and under-studied in low-income rural areas where different conditions may result in different transmission risks. In two low-income rural communities near the coast of Ecuador, we detected and characterized leptospira from febrile patients and putative animal reservoirs. Our results show the complexity of leptospirosis epidemiology and suggest that livestock may serve as an under recognized source of leptospirosis. Understanding the epidemiology of leptospirosis in many settings provide insights into transmission that can ultimately be used to to prevent and control this disease.

## Introduction

Leptospirosis is an infectious disease that causes morbidity in about 1.03 million people each year [1]. Severe disease produces multisystem complications such as acute renal or hepatic failure, or severe pulmonary hemorrhage that can lead to death [2,3]. Leptospirosis has a widespread distribution, but is mostly prevalent in tropical and poor regions of the world [1].

The causative agents of this zoonotic disease are spirochete bacteria in the genus *Leptospira*. Bacteria are shed into the environment via urine from infected animals and are transmitted to humans through skin abrasions or mucosal surfaces by direct contact with urine or with contaminated environmental sources [3,4]. Rats are thought to play a major role in human infection, and are considered the main reservoir of leptospirosis in urban slums [5,6]. High rat density near houses, living or working near garbage, exposure to open sewers, and others risk factors have been identified for leptospirosis [7,8]. Likewise, exposure to infected livestock is a recognized risk factor for leptospirosis, but is usually considered an occupational hazard. Leptospirosis infection is also associated with exposure to contaminated environmental sources such as during flooding events, working in rice or cane fields, or when exposed to river water during recreational activities [4,9–12].

Leptospirosis in low-income rural areas has been under-reported and under-studied, resulting in large knowledge gaps regarding disease transmission in such places, despite the fact that a large portion of the human population lives in rural areas. Differences in ecology and human behavior between these communities and urban slums, such as animal and pathogen diversity, livestock ownership and recreational activities, might drive differences in the epidemiology of this disease. Here, we present the findings of a two year diversity and prevalence study conducted in rural Ecuador. We collected samples from human febrile patients, cattle, pigs and rats to test for the presence of infectious *Leptospira* spp. using a newly developed sensitive and specific PCR assay We also genotyped positive samples using an amplicon sequencing approach that allowed us to identify the distribution of genotypes across potential animal reservoirs in an attempt to link host species (and exclude others) to human disease.

## Materials and Methods

### Study site

Our study was carried out from December 2013 to June 2015, and took place in two rural sites in Manabi Province, Ecuador (S1 Fig). Site 1: Abdon Calderon (1° 2' 0" S, 80° 20' 0" W), a rural parish near to Portoviejo City. Site 2: the rural communities of Ayacucho, Honorato Vasquez and, La Union Pueblo Nuevo in Santa Ana de Vuelta Larga Parish (1°12′25″S 80°22′15″W). The distance between Site 1 and Site 2 is approximately 9 Km. The economy of these rural communities is primarily based on agriculture, but subsistence animal ownership is common with animals living in close proximity to houses. Abdon Calderon and Santa Ana de Vuelta Larga were chosen for this study due to the high leptospirosis prevalence recorded by the Ecuadorian Health Ministry in 2012.

### Livestock and rat sampling

Urine samples from cattle and pigs raised in Calderon (Site 1) and Santa Ana (Site 2) were collected from local slaughterhouses. In these communities, animal owners (or their friends or neighbors) take their livestock to the slaughterhouses and were able to verify the origin of each animal sample. We visited the slaughterhouses twice a month and the number of samples received ranged from 0 to 10, depending on the presence of animals from each site (S1 Table). Samples from 48 cattle and 35 pigs were obtained from Site 1, and 117 cattle and 93 pigs from Site 2 slaughterhouses. Urine was collected directly from bladders of slaughtered animals and transported on ice to our laboratory in Quito, where they were stored at -20°C. Rat snap traps were provided to willing homeowners who were asked to set the traps inside their homes every 15 days. Homeowner’s efforts were likely sporadic and we were notified when trapping was succesfull. Kidneys were obtained from 36 and 65 rats (*Rattus* spp.) from Site 1 and Site 2 respectively, and placed in 90% molecular grade ethanol. DNA was extracted from rat tissue samples within a week of collection.

### Human samples

Blood and urine samples were obtained from febrile patients (1–6 days of fever) with no diarrheic or acute respiratory symptoms presenting at Site 1 and Site 2 local Public Health Centers. All samples were collected by the Health Centers for routine diagnostic testing targeting other diseases such as Dengue, Chikungunya, and urinary tract infections. Aliquots of residual samples were stored at -20°C. Throughout a period of 18 months, paired samples (urine and sera) from 449 patients were collected at the Site 1 health center. At the Site 2 health center, paired samples were collected from 149 patients while single serum (n = 72) and urine (n = 10) samples were collected from 82 patients.

### Culture in EMJH media

One or 2 drops of fresh human blood were injected in a rubber stopper tube (for the blood collection) which contained 7 mls of EMJH semi-solid medium (supplemented with 200 μg/mL 5- fluorouracil) and incubated for 1 or 2 weeks at room temperature after which the tubes were transported to the main laboratory where 1ml of the primary culture was inoculated in a new tube containing 7 mls of semi-solid EMJH medium; tubes were incubated for another 4 months at 30°C and monitored for leptospiral growth using a dark-field microscopy. Animal urine was collected directly from bladders at slauterhouses with a syringe, and 1 ml was inoculated into a tube containing 10 mls of liquid EMJH, tubes were inmediatelly transported to the main laboratory (10 hours) at 4°C and subjected to 3 10-fold dilutions in semisolid EMJH medium supplemented with 200 μg/mL 5- fluorouracil. Cultivation was attempted with a total of 56 human blood, 86 pig urine, and 123 cattle urine samples.

### DNA extraction of animal and human samples

DNeasy Blood and Tissue kit (Qiagen, CA, USA) was used to extract DNA from 200 uL of human sera and from leptospira EMJH isolates. Animal urine and rat kidney DNA was extracted as previously described [13]

### Assay design: Target sequence selection

We searched the 278 publicly available leptospira 16S rRNA gene sequences in the NCBI (http://www.ncbi.nlm.nih.gov) and JGI (https://img.jgi.doe.gov) databases for phylogenetically informative signatures among *Leptospira* spp. We used MEGA 6 [14] to align complete 16S rRNA gene sequences and identified single nucleotide polymorphisms (SNP) that provided discriminatory power among and within “pathogenic”and “intermediate” clades. Such SNP signatures were identified in a 153 bp region of the gene. This region corresponds to positions 3102729 to 3102577 and 1935913 to 1936065 in *Leptospira interrogans* serovar Lai str. 56609 (AE010300). For other pathogen species, TaqMan MGB assays have been used to detect very low amounts of target DNA and discriminate among species and even strains [15–18] and were therefore used here.

### Assay design: Positive control construction

In order to create positive controls and standardize quantification, a 330 bp fragment of the 16S rRNA gene from a “pathogenic” species (*Leptospira interrogans* Lai), and an “intermediate” species (*Leptospira licerasiae* VAR010) were synthesized as gBlocks gene fragments (IDT) and inserted inside the pCR 2.1 TOPO vector (Invitrogen Corp., Carlsbad, CA, USA). These fragments included the 153 bp fragment that provides identification and discrimination of “pathogenic” and “intermediate” leptospira (Table 1).

**Table 1:** Details of assays for detecting pathogenic and intermediate leptospira species. These two assays amplify the same region but the probes anneal to different targets within the amplified region. See also S3 Fig.

Assay	Primers	Probe	Fragment size	Group detected
**111**	F1: GAGTAACACGTGGGTAATCTTCCTR3: TTTACCCCACCAACTAGCTAATC	6FAM-CTGGGATAACTTT	153 bp	Pathogenic and intermediate leptospira
**50**		VIC-TCGGGTAAAGATT	153 bp	Pathogenicleptospira

### Assay design: Primer and probe design

We designed two single probe assays: (i) assay “111” detects all “pathogenic” and “intermediate”, but not “saprophytic” leptospira; (ii) assay “50” detects only “pathogenic” leptospira. TaqMan MGB probes and primers (Table 1) were designed using Primer Express Software (Life Technologies). Assays were designed as single probe assays that amplify the same region but probes anneal to different targets.

Both TaqMan MGB single-probe assays were run using a 7900HT Fast Real-Time PCR System (Applied Biosystems) with SDS v2.4 software. A total reaction volume of 10 μl was prepared by using 1x TaqMan Genotyping Master Mix (Applied Biosystems by Life Technologies, Foster City, CA, USA), 1μM of each primer, 300nM of each probe (Table 1), and 1μl of DNA. Thermal cycling conditions for the two assays (111 and 50) were as follows: 50°C for 2 min., 95°C for 10 min., followed by 45 cycles of 95°C for 15 sec., 58°C for 1 min.

### Assay design: Optimization and quantification

Assay metrics were determined by testing their performance across several parameters: accuracy, specificity, limit of quantification (LoQ) and detection (LoD), and linearity, as described in Price et al.[15]. SNP signatures used for probe design were subjected to both *in silico* (BLAST analysis) and laboratory screening to determine their accuracy towards leptospira species (S2 Table); all probes were specific to the target *Leptospira* clade. DNA from 15 leptospira species used in this study was supplied by the Royal Tropical Institute, Amsterdam, the Netherlands (*L*. *alexanderi*, *L*. *borgpetersenii*, *L*. *interrogans*, *L*. *kirschneri*, *L*. *kmetyi*, *L*. *noguchii*, *L*. *santarosai*, *L*. *weilii*, *L*. *fainei*, *L*. *inadai*, *L*. *licerasiae*, *L*. *wolffii*, *L*. *biflexa*, *L*. *vanthielii*, *L*. *wolbachii*). Non- leptospira species were tested to determine accuracy of assays towards leptospira species. These species were *Acinetobacter baumanii*, *Klebsiella pneumoniae*, *Staphylococcus epidermidis*, *Escherichia coli*, *Enterococcus faecalis*, *Enterobacter aerogenes*, *Moraxella catarrhalis*, *Streptococcus agalactiae*, *Neisseria meningitides*, and *Listeria monocytogenes* (S2 Table), and were chosen because they belong to genera that are either common pathogens or commonly associated with humans and other animals. For laboratory testing, the *Leptonema illini* 16S rRNA complete gene (chosen because it is the nearest neighbor of *Leptospira* genus) was synthetized by gBlocks gene fragments (IDT) and inserted inside the pCR 2.1 TOPO vector (Invitrogen Corp., Carlsbad, CA, USA) to keep it stable.

### Quantification

Known quantities of control vectors (see positive control construction) were used for determination of lowest LoQ (S2 Table) and LoD (S3 Table). The lowest LoQ was defined when 4 of 4 replicates amplified with a cycle threshold (C_T_) of <0.3 standard deviation from the mean C_T_. The lower LoD was measured, after defining the lowest LoQ, as the lowest concentration of analyte that gave rise to signal (considering that negative controls gave no signal). Range of linearity of each assay was determined by 10-fold dilutions that resulted in a Ct separation of about 3.4. Loss of linearity was defined as the lowest dilution point where this separation was seen. We also tested robustness of the assays by varying the annealing temperature from 56°C to 60°C.

### Sample analysis

All samples were tested with assays 111 and 50, additionally and in order to test PCR inhibitor compounds that may lead to false-negative results, we amplified a fragment of the beta-actin gene [19] in ten percent of human and animal samples, this fragment is expected to be present in all pig, rat, cattle and human tissue. No PCR inhibition was found. Amplicons of all positive samples for assays 111 and 50 were diluted to 10^5, and re-amplified using the same forward and reverse primers (Table 1) but containing a universal tail for indexing [20]. Briefly, primers for specific amplicons were designed in order to be compatible with Illumina adapter sequences, amplicons were reamplified in a conventional thermocycler with the following conditions: 94°C for 5 min., followed by 15 cycles of 94°C for 30 sec., 60°C for 30 sec., 72°C for 2 min., and a final extension at 72°C for 5 min. Amplicons were pooled and sequenced with the Illumina MiSeq in order to confirm the results of the assays and identify leptospira species present in each sample. Raw sequences were demultiplexed with the MiSeq software and also with the fastq-multx tool from ea-util package [21]. Consensus sequences were obtained using DNASTAR Seqman software for sequence assembly and contig management. For pathogenic (and intermediate) species, sequences matched a single species with 100% identity, providing confidence in our species assignments (S2 Fig).

When samples were available, serum samples from patients positive for PCR (*Leptospira* DNA in urine or sera) were tested for IgM antibodies using the Panbio Leptospira IgM ELISA (Queensland, Australia). MAT (Microscopic Aglutination Test) was also performed on these samples at the national reference laboratory: Instituto Nacional de Salud Pública e Investigación, Guayaquil-Ecuador.

### Phylogenetic analysis

Phylogenetic reconstruction of amplicon sequencing results was carried out in MEGA6 [14] using the Maximum Likelihood method, with the Kimura 2-parameter model [22]. This model had the lowest Bayesian Information Criterion score and corrected Akaike information criterion as determined through model selection analysis in MEGA. Cluster confidence was determined by performing 100 bootstrap replicates.

### Statistical analysis of rainfall and leptospirosis associations

Association analysis of leptospirosis cases (n = 227) and rainfall from Site 1 (Calderon-Manabi) was performed using data from the INHAMI (National Institute of Meteorology and Hidrology) data-base (http://www.inamhi.gob.ec/) and serologically confirmed leptospirosis cases provided by the Ecuadorian Health Ministry (MSP-VGVS-2015-0197-O). Only data from Site 1 was used as meteorological data from Site 2 were not available. For this, we defined high and low precipitation thresholds using local rainfall data; we defined a month with low precipitation as accumulated rainfall of ≤50 mm and a month of high rainfall with precipitation >50 mm. In order to account for the time between infection and clinical onset of the disease, monthly leptospirosis cases were matched with precipitation values from the same and previous months. Association between variables was performed in the SPSS program by running a Generalized Linear Equation model using the Wald statistic and the Maximum likelihood estimate. Significant differences among all pairs were identified using Fisher's exact test. Homogeneity of proportions was tested with *post hoc* pair-wise comparisons [23]. P-values were adjusted with the Benjamini & Hochberg method [24], and the odds ratio probability was calculated by using the Epitools package in R [25,26].

### Ethics statement

Human samples were collected by Ecuadorian Health Ministry Technicians and analyzed with the authorization of The Bioethical Committee of Universidad San Francisco de Quito (study code 2011–40) and by the Northern Arizona University Institutional Review Board (482212–1). Cattle and pig urine samples were collected from animals slaughtered for human consumption at the local abattoir and were thus processed as normal work of the abattoir. Verbal consent from the animal owners was provided. *Rattus spp*. were collected by homeowners using methods consistent with AVMA guidelines for euthanasia and approved by the NAU IACUC (Protocol 13–006).

## Results

### Performance of TaqMan PCR assays

Real-time PCR assays described here can detect infectious leptospira species (excluding “saprophytic” species) and can discriminate among members of the “pathogenic” and “intermediate” clades. As predicted by *in silico* analysis, assays 111 and 50 were 100% specific for species within the “pathogenic” and “intermediate” clade, and for the “pathogenic” species, respectively. Both assays 111 and 50 must be used to define species in the “intermediate” clade; assay 50 is needed to exclude “pathogenic” species from those detected using Assay 111. None of the saprophytic leptospira species, the 10 non- leptospira species, or the closely related *Leptonema illini* amplified with either assay (S1 Table). Both assays exhibited the lowest limit of quantification (LoQ) and lowest limit of detection (LoD) to be one 16S rRNA copy per microliter of extracted DNA (S3 Table). Range of linearity of assays 111 and 50 was 10^8 to10^1 16S rRNA genes. Assay 111 is robust along a 4°C variation in annealing temperature (56–60°C) and assay 50 provided robust amplification along a 2°C variation in annealing temperature (56–58°C).

### Leptospira species identification

Amplicon sequencing also enabled separation of the main “pathogenic” and “intermediate” clades but provided topological resolution within these clades similar to that provided by others who used a 1,230 bp fragment (Levett et al. 2015) to describe the molecular phylogenetics of the *Leptospira* genus (S2 Fig). Sequencing this 153 bp region allowed us to discriminate among most of the species of leptospira found in animal urine, rat kidney, and human urine and sera samples. The sequences obtained matched 16S sequences from known species at 99–100% (S6 Table).

### Leptospira detection in humans and animals

From Site 1, samples from febrile patients with no diarrhea or acute respiratory symptoms were positive for pathogenic leptospira DNA in 17.3% (n = 78/449) of the samples: sera 2%, (n = 8/449), and urine 15.6% (n = 70/449). Site 2 patients showed positivity in 9.5% (n = 22/231): 3.6% in sera (n = 8/219), and 8.8% in urine (n = 14/159). We never detected *Leptospira* DNA in sera and urine from the same patient.

**Fig 1:**
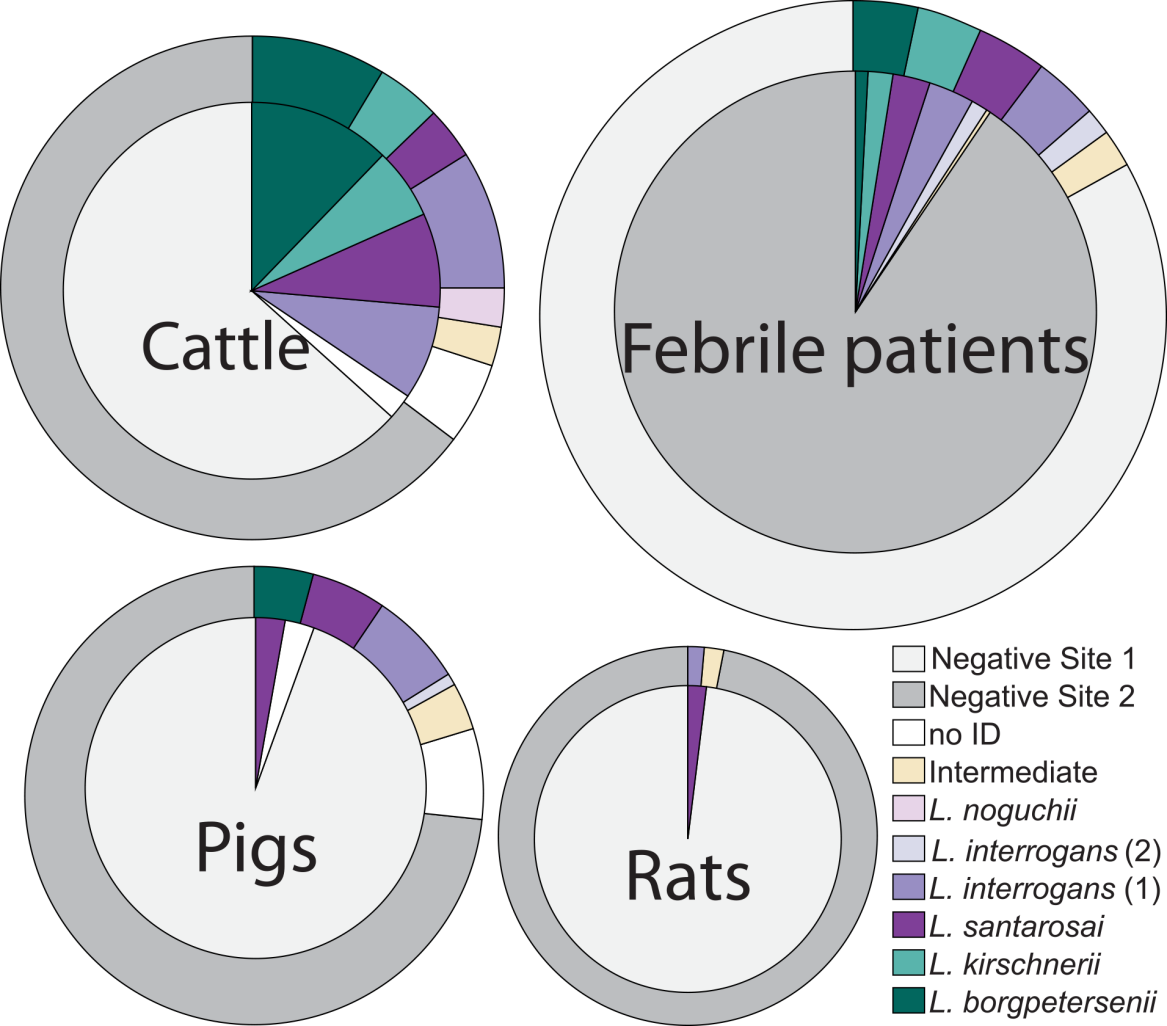
Leptospira genotyping results in febrile patients, cattle, pigs and rats sampled at our study sites. Concentric circles represent the sample size in Site 1 (colored with light gray) and Site 2 (dark gray). The order of these concentric circles is dependent on the sample size at each site. The proportion of each *Leptospira* genotype is marked with a different color, however white portions represent the percentage of samples for which genotyping was unsuccessful. Sample sizes for each group is detailed in Table 2 and S6 Table.

Urine from slaughtered cattle and pigs, and rat kidney were collected in both sites over the same time period when human samples were obtained (Fig 1). Positivity in cattle urine was 35.4% (n = 17/48) and 35.9% (n = 42/117) for Site 1 and Site 2, respectively. Lower positivity was found in pig urine: 5.7% (n = 2/35) for Site 1 and 26.9% (n = 25/93) Site 2. Rat kidney positivity was low for both sites: 2.8% (n = 1/36) in Site 1, and 3.1% (n = 2/65) in Site 2 (Table 2). Additionally, we recovered leptospira isolates from 1 out of 56 (*L*. *santarosai*) human blood samples and 2 (*L*. *interrogans*) out of 123 cattle urine; drafts of these genomes were deposited in GenBank [27].

**Table 2:** *Leptospira* DNA positivity in samples collected in 2014 and the first 6 months of 2015 from two rural communities in Manabi-Ecuador.

**SITE 1**	**Positive**	**Total**	**%**
Human urine	70	449	15.6
Human sera	8	394	2.0
Cows	17	48	35.4
Pigs	2	35	5.7
Rats	1	36	2.8
**SITE 2**	**Positive**	**Total**	**%**
Human urine	14	159	8.8
Human sera	8	219	3.7
Cows	42	117	35.9
Pigs	25	93	26.9
Rats	2	65	3.1

### Leptospira diversity

Six different leptospira species were identified in both study sites: *L*. *borgpetersenii*, *L kirschnerii*, *L santarosai*, *L*. *interrogans*, *L noguchii*, and an intermediate species within the *L*. *licerasiae* and *L*. *wolffii* clade (Fig 1 and S4 Table). Our amplicon sequencing data divide *L*. *interrogans* into two genotypes. Despite Site 1 being 9 km away from Site 2, the frequency of leptospira species circulating within each site did not differ between sites (χ-squared = 9.89, adj.p-value = 0.27) or between 2014 and 2015 (χ-squared = 5.16, adj.p-value = 0.56). Additionally, there were no significant differences in the species of leptospira present in cattle and pigs (χ-squared = 10.06, adj.p-value = 0.124). We also found that *L*. *interrogans* and *L*. *borgpetersenii* are more likely to be carried by cattle (β > 1.08, p-value < 0.005, β > 1.25, p-value < 0.005).

We also characterized the serological diversity on some febrile patient samples that were collected one to six days after the onset of fever. Thirteen of 16 DNA-positive sera samples and 74 of 84 DNA-negative sera samples from patients who had DNA-positive urine were tested with a commercial leptospira IgM ELISA and with MAT by the local reference laboratory. ELISAs of all patients with DNA-positive sera samples (representing acute infections) were negative and only one was MAT positive to serovar Canicola. As such, we were not able to determine the rate of false negative detection of PCR methods. Results on DNA-negative sera samples from febrile patients with DNA-positive urine also showed low positivity; only 3 were positive for ELISA and 20 for MAT.

### Rainfall and leptospirosis

Association analysis using leptospirosis IgM positivity recorded by the Health Ministry for 2011–2014 from Site 1 and monthly rainfall showed that 30.9% of positive sample variation could be explained by rainfall in the same month (R^2^_2011-2014_ = 0.309, CI: 0.027–0.545, p-value = 0.032). In contrast, 57% of variation in positive cases could be explained by rainfall from the preceding month (R^2^_2011-2014_ = 0.57, CI:0.36–0.75, p-value = 1.18 x 10^−5^). For each year, the percentage of cases explained by rainfall in the same month ranged from 12–61% (R^2^_2011_ = 0.56, CI: -007-0.8, p-value = 0.05; R^2^_2012_ = 0.12, CI: -0.4–0.64, p-value = 0.7; R^2^_2013_ = 0.51, CI: -0.095–0.83, p-value = 0.090; R^2^_2014_ = 0.61, CI:0.024–0.9, p-value = 0.044). In contrast with the percentage of cases explained by rainfall in the preceding month that ranged from 51–69% (R^2^_2011_ = 0.67, CI: 0.11–0.9, p-value = 0.024; R^2^_2012_ = 0.58, CI: 0.012–0.86, p-value = 0.047; R^2^_2013_ = 0.51, CI: -0.095–0.83, p-value = 0.093; R^2^_2014_ = 0.69, CI:0.199–0.9, p-value = 0.0123).

Analysis of the presence of *Leptospira* DNA in urine and serum samples from all febrile patients who presented to the local health center between 2014 and the beginning of 2015 showed that the odds of a febrile patient being infected with *Leptospira* was not higher in months with high rainfall (OR_Site 1_ = 0.95, CI: 0.55–1.6, p-value = 0.85;OR_Site 2_ = 1.5, CI: 0.6–3.9, p-value = 0.4). Similar results were obtained when the number of cases per month was matched with precipitation from the previous month (Table 3).

**Table 3:** Association between rainfall and presence of *Leptospira* DNA in sera and urine febrile patients during the period of this study. Data were stratified into low precipitation (< 50 mm) and high precipitation months (>50 mm).

	Precipitation	Positive	Negative	TOTAL	Odds Ratio	CI	p-value
**SITE 1**	Current month						
	>50 mm	23	117	140	0.95	0.548–1.614	0.8
	≤50 mm	53	256	309			
	Previous month						
	>50 mm	17	92	109	0.88	0.47–1.57	0.7
	≤50 mm	59	281	340			
**SITE 2**	Current month						
	>50 mm	12	109	121	1.23	0.49–3.17	0.6
	≤50 mm	9	101	110			
	Previous month						
	>50 mm	9	72	81	1.44	0.56–3.6	0.4
	≤50 mm	12	138	150			

## Discussion

Very little is known about leptospirosis in Ecuador or the epidemiology of leptospirosis in low-income, rural communities. The aim of our study was to better understand some of the factors thought to influence occurrence and transmission of pathogenic leptospira in these areas. These factors include animal reservoirs and rainfall.

Detection of pathogenic leptospira is difficult due to the practical complications of culturing, the diversity of hosts, genetic diversity of the pathogen, and heterogeneous or absent symptoms [3,28,29]. Molecular detection of pathogenic leptospira is, in general, more sensitive than culture or the microscopic agglutination tests (MAT) [28,30]. However, currently used molecular detection assays suffer from suboptimal sensitivity and inability to discriminate between species in the “pathogenic” and “intermediate” clade [31–37]. Recently, species in the “intermediate” clade have been associated with disease [37–40], presenting the need to include them in epidemiological studies. Moreover, “intermediate” leptospira have been shown to infect animals and humans in Ecuador [13]. The methodology presented here allowed us to detect and discriminate between DNA from “pathogenic” and “intermediate” leptospira species in clinical and animal samples.

In contrast with previous reports of rats being the main reservoirs of leptospirosis in urban slums, [5,6,41–43], our results show low positivity in rats but high positivity in cattle (Table 2). This suggest that in our study sites and during our sampling period, cattle are likely to be more important reservoirs for leptospirosis than rats.

Leptospirosis is commonly associated with rainfall [9,44]. Regression analysis from serological data on human cases of leptospirosis collected by the Health Ministry for 2011–2014 showed a strong association with rainfall. Interestingly, our analyses of *Leptospira* DNA-positive results from sera and urine samples collected at this same site between 2014 and the beginning of 2015 show no such association. Our 16 DNA-positive sera samples, however were collected in the rainy season (n = 15) or in the following month (n = 1). In contrast, the 84 DNA-positive urine samples show no association with rainfall, possibly due to our inability to attribute infection to a specific month. Detection of *Leptospira* DNA in urine and not serum is likely due to sampling after the bacteria have been cleared from the bloodstream. *Leptospira* is not typically detected in serum after 5 days of fever, but can be found in urine months later [4]. Antibodies can be detectable by MAT before and after bacteria are cleared from circulation, thus paired samples showing a temporal increase in titer indicate acute cases [4]. Of the 84 DNA-positive urine samples, MAT analyses were conducted on 74 and 20 (27%) were MAT positive. As convalescent samples are never obtained by the Health Ministry, these results do not allow us to discriminate between acute and long-term disease. Conversely, 54 of these samples (73%) were MAT negative, indicating chronic carriage (assuming that present serovars can be adequately detected with the serological test). As chronic leptospirosis is usually asymptomatic [45,46], we suspect that the fever of these patients and presentation at the clinic might have been due to another illness. Overall, our results suggest that rainy seasons are important for acute infections, but also suggest that chronic carriage is common, the implications of which deserve further exploration.

Previous work on leptospirosis epidemiology suggests that some serotypes are more likely to infect certain hosts [29,47,48], therefore the genetic diversity in the population of hosts may also be species specific. Thus, finding the same species in both rats and humans [39] may provide evidence for an epidemiological link. In contrast, our genotyping work shows that different host species are routinely infected by a variety of circulating leptospira species and these leptospira were not differentially associated with certain host species. Importantly, all sampled animal types contained leptospira species that were also associated with human clinical samples, similar to what has been reported in Southeast Asia [36]. This suggests that genotype associations (at a species level of resolution) alone cannot be used to exclude any of these animal types as potential contributors to human transmission. Higher resolution genotyping methods and epidemiological information such as evidence of direct contact with a given animal would be needed in addition to genotype information to establish an epidemiological link. Given the high prevalence in cattle and their shared genotypes with humans, we hypothesize that cattle are playing an important roll in the transmission to humans. However other animals also share genotypes with humans.

Distribution and morbidity of leptospirosis depends on geographic and socio-economic features [1]. Therefore differences between urban and rural leptospirosis should be expected. Our study provides strong evidence that suggest that leptospirosis in low-income rural communities might be different than in urban slums. People living in low-income rural areas usually share their peri-domestic and recreational environment with livestock and in some cases with sylvatic animals. This contrasts with urban areas where contact with these animals is mostly encountered in occupational settings. Our study also exhibits the importance of considering other animals besides rats as important reservoirs of the disease. It also suggests that some rural areas might have higher diversity of pathogenic leptospira species than what has been shown for urban-slums. We believe that it is important to understand leptospirosis ecology in low-income rural areas, especially considering the high burden of leptospirosis in rural areas [1], and that in Latin America, 30–60% of people live in rural areas [49].

## Supporting Information

S1 Checklist STROBE Checklist. (DOC)

S1 Fig Study sites located near the coast in Manabi Province, Ecuador. (DOCX)

S2 Fig Molecular phylogenetic analysis of Leptospiraceae using a 153 bp fragment of the 16S rRNA gene that corresponds to the region amplified with the F1-R3 primers. (DOCX)

S3 Fig Sequence alignment of one representative strain for each species with the position where primers (R3 and F1) and probes (111 and 50) anneal. (DOCX)

S1 Table Animal and human sample details. (XLSX)

S2 Table Determination of assay accuracy and specificity by *in silico* comparisons and TaqMan real-time PCR results. (DOCX)

S3 Table Establishing the Lowest limit of quantification of 111 and 50. (DOCX)

S4 Table Establishing the LoD (limit of detection) of 111 and 50 assays with 11 replicates. (DOCX)

S5 Table Amplicon sequences of leptospira genotypes. (DOCX)

S6 Table Leptospira genotypes identified after amplicon sequencing of positive samples. (XLSX)
