# Supplementary material for: High Leptospira Diversity in Animals and Humans Complicates the Search for Common Reservoirs of Human Disease in Rural Ecuador
Source: PLoS Negl Trop Dis. 2016 Sep 13;10(9):e0004990. doi: 10.1371/journal.pntd.0004990 (PMC5021363; doi:10.1371/journal.pntd.0004990)
Supplement: S1 Fig — (DOCX) [file pntd.0004990.s002.docx]

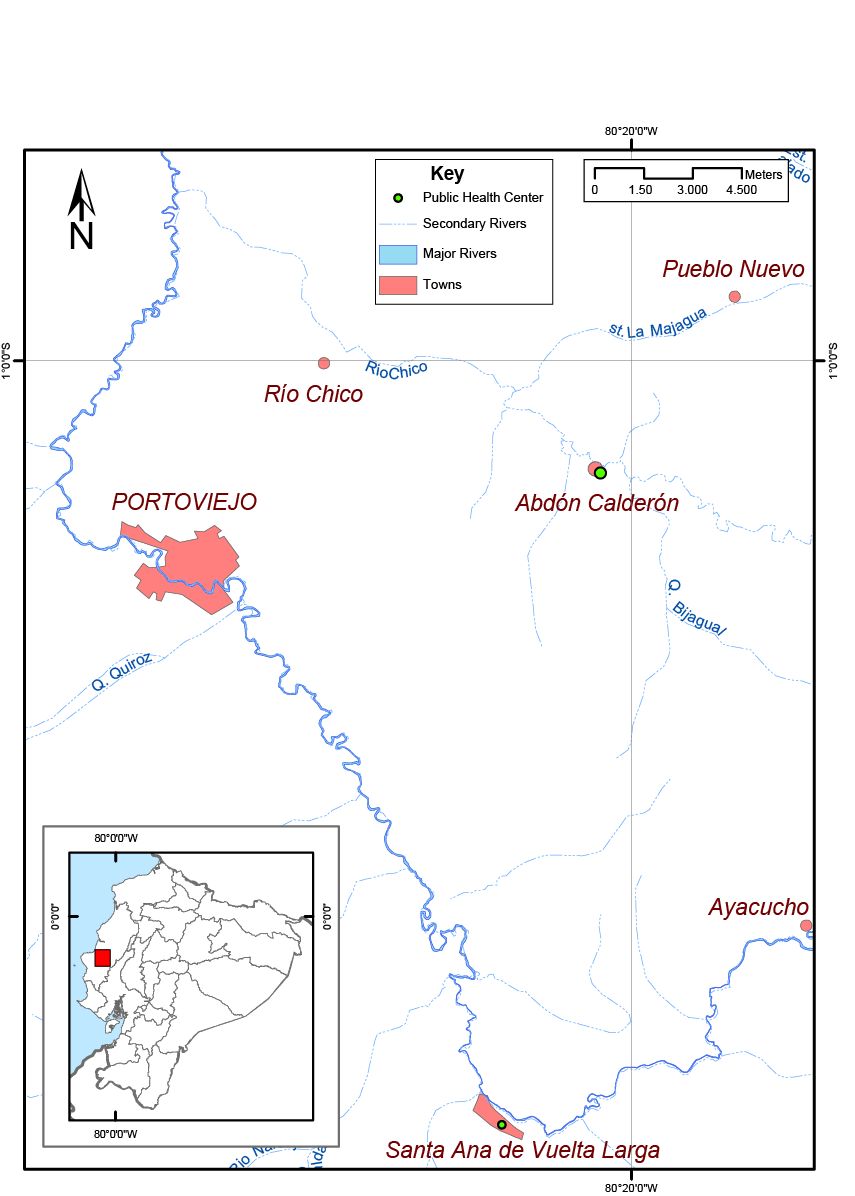


**S1 Figure**: **Study sites located near the coast in Manabi Province, Ecuador.** Site 1 is the community of Abdon Calderon and Site 2 is Santa Ana de Vuelta Larga, green circles indicate the location of the local public health centers.
