# Supplementary material for: High Leptospira Diversity in Animals and Humans Complicates the Search for Common Reservoirs of Human Disease in Rural Ecuador
Source: PLoS Negl Trop Dis. 2016 Sep 13;10(9):e0004990. doi: 10.1371/journal.pntd.0004990 (PMC5021363; doi:10.1371/journal.pntd.0004990)
Supplement: S2 Fig — (DOCX) [file pntd.0004990.s003.docx]

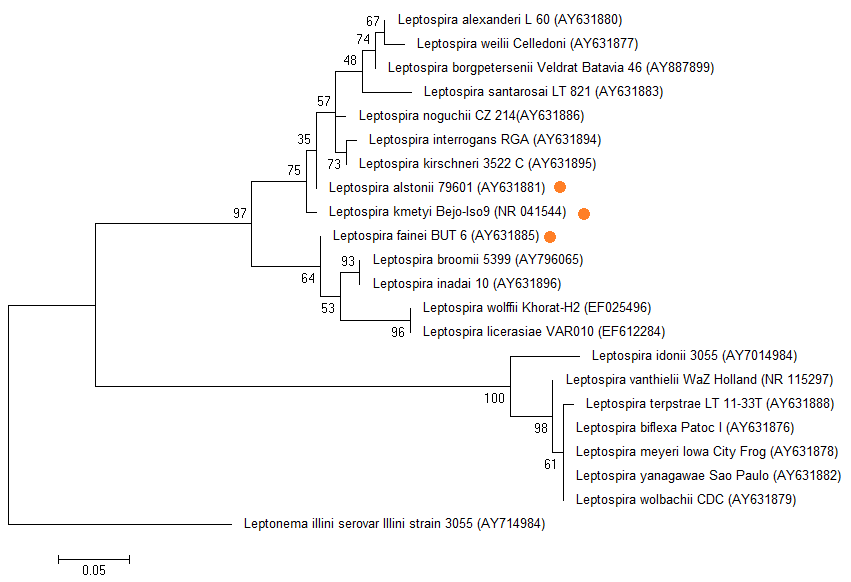


**S2 Figure: Molecular phylogenetic analysis of Leptospiraceae using a 153 bp fragment of the 16S rRNA gene that corresponds to the region amplified with the F1-R3 primers.** Tree was calculated using the maximum likelihood method based on the Kimura 2-parameter model with MEGA6. The percentage of trees in which the associated taxa clustered together is shown next to the branches. Orange dots show species that are located in slightly different positions than the tree published by Levett (2015).
