# Supplementary material for: High Leptospira Diversity in Animals and Humans Complicates the Search for Common Reservoirs of Human Disease in Rural Ecuador
Source: PLoS Negl Trop Dis. 2016 Sep 13;10(9):e0004990. doi: 10.1371/journal.pntd.0004990 (PMC5021363; doi:10.1371/journal.pntd.0004990)

**S3 Figure:** **Sequence alignment of one representative strain for each species with the position where primers (R3 and F1) and probes (111 and 50) anneal.**


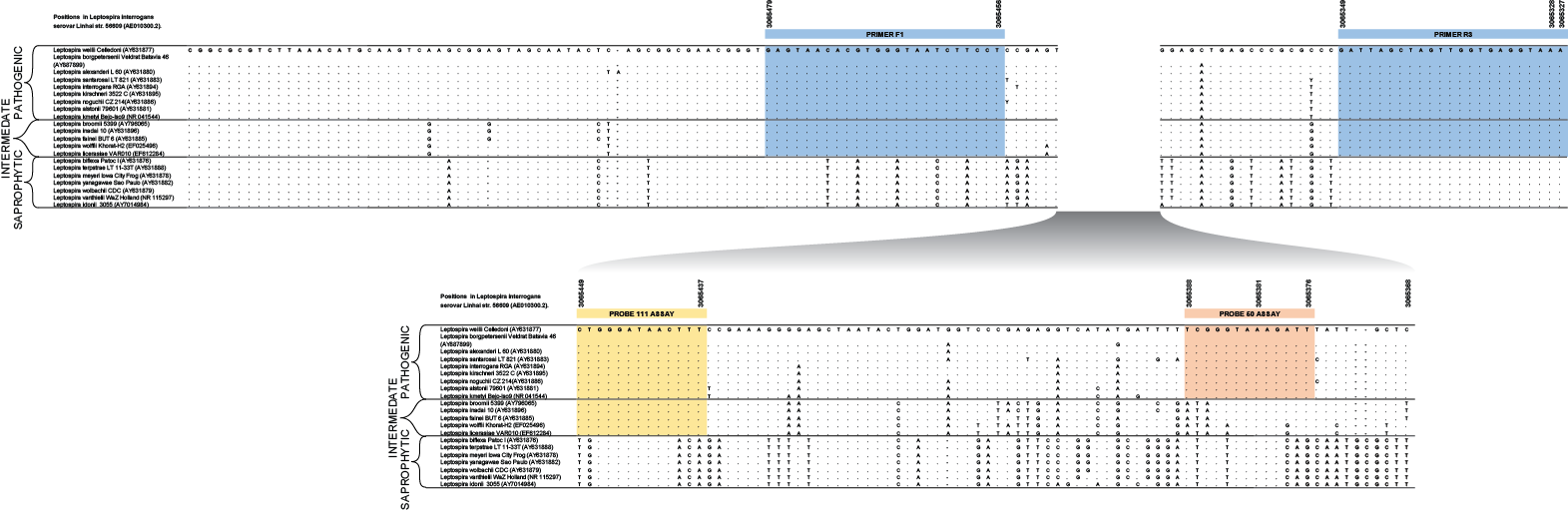

Supplement: S3 Fig — (DOCX) [file pntd.0004990.s004.docx]
