# Supplementary material for: High Leptospira Diversity in Animals and Humans Complicates the Search for Common Reservoirs of Human Disease in Rural Ecuador
Source: PLoS Negl Trop Dis. 2016 Sep 13;10(9):e0004990. doi: 10.1371/journal.pntd.0004990 (PMC5021363; doi:10.1371/journal.pntd.0004990)
Supplement: S2 Table — (DOCX) [file pntd.0004990.s006.docx]

**S2 Table:** **Determination of assay accuracy and specificity by *in silico* comparisons and TaqMan real-time PCR results.**
