# Supplementary material for: High Leptospira Diversity in Animals and Humans Complicates the Search for Common Reservoirs of Human Disease in Rural Ecuador
Source: PLoS Negl Trop Dis. 2016 Sep 13;10(9):e0004990. doi: 10.1371/journal.pntd.0004990 (PMC5021363; doi:10.1371/journal.pntd.0004990)
Supplement: S3 Table — (DOCX) [file pntd.0004990.s007.docx]

**S3 Table: Establishing the Lowest limit of quantification of 111 and 50.** ^a^Target 16S rRNA gene fragment sequence was inserted inside pCR®2.1 TOPO® vector (Invitrogen Corp., Carlsbad, CA, USA). ^b^Lowest LoQ (Lowest limit of quantification) was initially determined as the lowest amount of 16S rRNA copies at which all 4 replicates amplified (highlighted).

| **DNA** | **Average 16S rRNA gene copies^a^ per reaction** | **Assay 50 Lowest LoQ^b^** | **Assay 111 Lowest LoQ^b^** |
| --- | --- | --- | --- |
| *Leptospira interrogans* lai | 10^6 | 4 of 4 | 4 of 4 |
|  | 10^5 | 4 of 4 | 4 of 4 |
|  | 10^4 | 4 of 4 | 4 of 4 |
|  | 10^3 | 4 of 4 | 4 of 4 |
|  | 10^2 | 4 of 4 | 4 of 4 |
|  | 10^1 | 4 of 4 | 4 of 4 |
|  | 10^0 | 4 of 4 | 4 of 4 |
|  | 10^-1 | 2 of 4 | 3 of 4 |
|  | 10^-2 | 0 of 4 | 0 of 4 |
| *Leptospira licerasiae* VAR010 | 10^5 | 0 of 4 | 4 of 4 |
|  | 10^4 | 0 of 4 | 4 of 4 |
|  | 10^3 | 0 of 4 | 4 of 4 |
|  | 10^2 | 0 of 4 | 4 of 4 |
|  | 10^1 | 0 of 4 | 4 of 4 |
|  | 10^0 | 0 of 4 | 4 of 4 |
|  | 10^-1 | 0 of 4 | 3 of 4 |
|  | 10^-2 | 0 of 4 | 0 of 4 |
| *Leptospira biflexa* PATOC I | 10^5 | 0 of 4 | 0 of 4 |
|  | 10^4 | 0 of 4 | 0 of 4 |
|  | 10^3 | 0 of 4 | 0 of 4 |
|  | 10^2 | 0 of 4 | 0 of 4 |
|  | 10^1 | 0 of 4 | 0 of 4 |
|  | 10^0 | 0 of 4 | 0 of 4 |
|  | 10^-1 | 0 of 4 | 0 of 4 |
|  | 10^-2 | 0 of 4 | 0 of 4 |
| NTC | 0 | 0 of 10 | 0 of 10 |
