# Supplementary material for: High Leptospira Diversity in Animals and Humans Complicates the Search for Common Reservoirs of Human Disease in Rural Ecuador
Source: PLoS Negl Trop Dis. 2016 Sep 13;10(9):e0004990. doi: 10.1371/journal.pntd.0004990 (PMC5021363; doi:10.1371/journal.pntd.0004990)
Supplement: S4 Table — (DOCX) [file pntd.0004990.s008.docx]

**S4 Table: Establishing the LoD (limit of detection) of 111 and 50 assays with 11 replicates.** Lowest LoD was determined as the lowest amount of 16S rRNA copies at which 8 of 11 replicates amplified

| **50 ASSAY** |  |  |  |
| --- | --- | --- | --- |
| **DNA** | **Average 16S rRNA gene copies^a^ per reaction** |  | **replicates with signal** |
| *Leptospira interrogans* lai | 10^1 |  | 11 of 11 |
|  | 10^0 |  | 11 of 11 |
|  | 10^-1 |  | 8 of 11 |
|  | 10^-2 |  | 1 of 11 |
| *Leptospira licerasiae* VAR010 | 10^1 |  | 0 of 11 |
|  | 10^0 |  | 0 of 11 |
|  | 10^-1 |  | 0 of 11 |
|  | 10^-2 |  | 0 of 11 |
| *Leptospira biflexa* Patoc I | 10^1 |  | 0 of 11 |
|  | 10^0 |  | 0 of 11 |
|  | 10^-1 |  | 0 of 11 |
|  | 10^-2 |  | 0 of 11 |
| NTC |  |  | 0 of 11 |
| **111 ASSAY** |  |  |  |
|  |  |  |  |
| **DNA** | **Average 16S rRNA gene copies^a^ per reaction** |  | **replicates with signal** |
|  | 10^1 |  | 11 of 11 |
| *Leptospira interrogans* lai | 10^0 |  | 11 of 11 |
|  | 10^-1 |  | 8 of 11 |
|  | 10^-2 |  | 1 of 11 |
|  | 10^1 |  | 11 of 11 |
| *Leptospira licerasiae* VAR010 | 10^0 |  | 11 of 11 |
|  | 10^-1 |  | 8 of 11 |
|  | 10^-2 |  | 4 of 11 |
|  | 10^1 |  | 0 of 11 |
| *Leptospira biflexa* Patoc I | 10^0 |  | 0 of 11 |
|  | 10^-1 |  | 0 of 11 |
|  | 10^-2 |  | 0 of 11 |
| NTC |  |  | 0 of 11 |
|  |  |  |  |
