# Supplementary material for: High Leptospira Diversity in Animals and Humans Complicates the Search for Common Reservoirs of Human Disease in Rural Ecuador
Source: PLoS Negl Trop Dis. 2016 Sep 13;10(9):e0004990. doi: 10.1371/journal.pntd.0004990 (PMC5021363; doi:10.1371/journal.pntd.0004990)
Supplement: S5 Table — (DOCX) [file pntd.0004990.s009.docx]

**S5 Table.** Amplicon sequences of leptospira genotypes.

| Sequence | Match with GenBank sequence, % identity |
| --- | --- |
| L. borgpetersenii;  TTACCTCACCAACTAGCTAATCGGGCGCGGGCTCATCTCCGAGCAATAAATCTTTACCCGAAAAATCCTATGATCTCTCGGGACTATCCAGTATTAGCTCCCCTTTCGGAAAGTTATCCCAGACTCGGAGGAAGATTACCCACGTGTTACTC | CP015052.1, 99% |
| L. kirschnerii;  CTTTACCTCACCAACTAGCTAATCGGACGCGGGCTCATCTCCGAGCAATAAATCTTTACCCGAAAAATCTTATGATCTCTCGGGACCATCCAGTATTAGCTTCCCTTTCGGAAAGTTATCCCAGACTCGGAGGAAGATTACCCACGTGTTACTC | KP125531.1, 100% |
| L. santarosai^*^;  CTTTACCTCACCAACTAGCTAATCGGACGCGGGCTCATCTCCGAGCAATGAATCTTTACCCGATACATCCTATGATCTATCGGGACTATCCAGTATTAGCTCCCCTTTCGGAAAGTTATCCCAGACTCGAAGGAAGATTACCCACGTGTTACTC | KX008568.1, 100% |
| L. interrogans 1^*^;  CTTTACCTCACCAACTAGCTAATCGGACGCGGGCTCATCTCCGAGCAATAAATCTTTACCCGAAAAATCTTGTGATCTCTCGGGACCATCCAGTATTAGCTTTCCTTTCGAAAAGTTATCCCAGACTCGGAGGAAGATTACCCACGTGTTACTC | LSSQ00000000, 100% |
| L. interrogans 2;  CTTTACCTCACCAACTAGCTAATCGGACGCGGGCTCATCTCCGAGCAATAAATCTTTACCCGAAAAATCTTATGATCTCTCGGGACCATCCAGTATTAGCTTCCCTTTCGGAAAGTTATCCCAGACTCAGAGGAAGATTACCCACGTGTTACTC | CP013147.1, 100% |
| L. noguchii;  CTTTACCTCACCAACTAGCTAATCGGACGCGGGCTCATCTCCGAGCAATCAATCTTTACCCGAAAAATCTTATGATCTCTCGAGACCATCCAGTATTAGCTTCCCTTTCGGAAAGTTATCCCAGACTCGGAGGAAGATTACCCACGTGTTACTC | AY461882.1, 99% |
| L. licerasiae/L. wolffii;  TTACCTCACCAACTAGCTAATCGGCCGCGGGCTCATCTCCGAACAGTAAACCTTTATCTATCAAATCCTGTGATCCAATAGAACTATCCGGTATTAGCTTTCCTTTCGGAAAGTTATCCCAGATTCGGAGGAAGATTACCCACGTGTTACTC | EF612287.1, 100%  KM259910.1, 100% |

^*^ These sequences match whole genome sequences from GenBank: accession numbers: LSSR00000000 for *L santarosai*, and LSSQ00000000 for *L. interrogans*1 ([1](#_ENREF_1)).

1. Barragan V, Sahl JW, Wiggins K, Chiriboga J, Salinas A, Cantos NE, et al. Draft Genome Sequence of the First Pathogenic Leptospira Isolates from Ecuador. Genome announcements. 2016;4(3).
